# Exploring *absent* protein function in yeast: assaying post translational modification and human genetic variation

**DOI:** 10.15698/mic2021.08.756

**Published:** 2021-07-02

**Authors:** Christina S. Moesslacher, Johanna M. Kohlmayr, Ulrich Stelzl

**Affiliations:** 1 Institute of Pharmaceutical Sciences and BioTechMed-Graz, University of Graz, Graz, Austria.; # Contributed equally to the writing of this review.

**Keywords:** yeast two-hybrid, deep mutational scanning, massively parallel reporter assays, protein-protein interaction, cancer mutations, phosphorylation, DNA methylation

## Abstract

Yeast is a valuable eukaryotic model organism that has evolved many processes conserved up to humans, yet many protein functions, including certain DNA and protein modifications, are *absent*. It is this absence of protein function that is fundamental to approaches using yeast as an *in vivo* test system to investigate human proteins. Functionality of the heterologous expressed proteins is connected to a quantitative, selectable phenotype, enabling the systematic analyses of mechanisms and specificity of DNA modification, post-translational protein modifications as well as the impact of annotated cancer mutations and coding variation on protein activity and interaction. Through continuous improvements of yeast screening systems, this is increasingly carried out on a global scale using deep mutational scanning approaches. Here we discuss the applicability of yeast systems to investigate *absent* human protein function with a specific focus on the impact of protein variation on protein-protein interaction modulation.

## INTRODUCTION

Yeasts, in particular *Saccharomyces cerevisiae* and *Schizosaccharomyces pombe*, are key eukaryotic model organisms in molecular and cell biology research. As basic unicellular eukaryotic organisms they rely on many molecular pathways, cellular functions and biological processes, that are at their core often conserved up to humans. With 4,000 homologous genes between yeast and human, many insights into human biology are drawn from studying yeast and from approaches to humanize yeast functions. As outlined in recent reviews [1, 2], the latter can be done to various degrees, from studying genes and processes in yeast that mechanistically resemble molecular events in human, complementing loss of yeast protein function by human proteins [3] to humanizing entire pathways [4].

At the same time yeast is lacking functions that have evolved in higher eukaryotes such as vertebrates and mammals only. This includes all features of multicellularity and its developmental aspects, yet also other cellular functions, regulatory events, biochemical activities and cellular processes that are not present in yeast (here referred to as “*absent*”). When introducing proteins and functions from higher organisms that are naturally *absent* in lower eukaryotes, they may be active in the heterologous cellular context, irrespectively of the fact that yeast has not evolved the function and cannot process the molecular signals accordingly. For example, ectopically expressed full-length epidermal growth factor receptor (EGFR), a human receptor tyrosine kinase, is active in yeast without generating a functional molecular signaling output. However, combined expression with human GRB2 and human SOS, the most upstream components of mammalian MAPK signaling, activates the yeast Ras/cAMP pathway in a *CDC25/YLR310C* deficient *S. cerevisiae* strain [5]. While these experiments demonstrate that EGFR can be active in yeast, in most cases these heterologous human protein activities appear functionally isolated in a living organism which cannot regulate or process the activity further.

**Figure 1:**
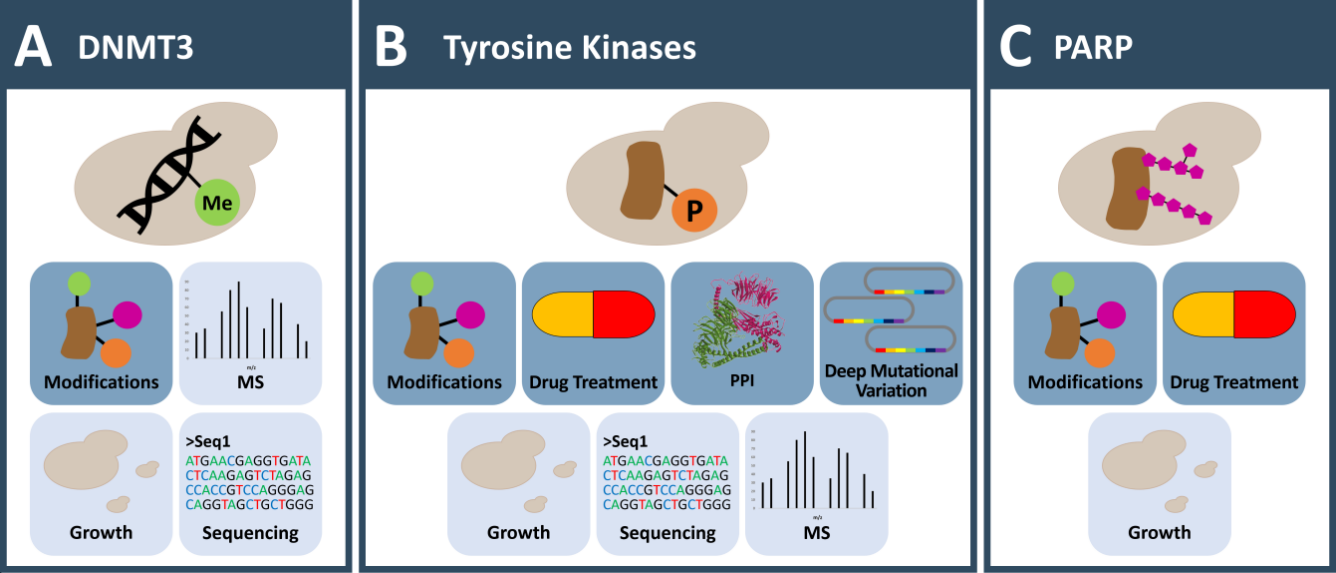
FIGURE 1: Applications to investigate DNA methylation and post translational protein modifications otherwise *absent* in yeast. **(A)** Yeast is used as a model organism to investigate cytosine DNA methylation induced *de novo* via human DNMTs. A special focus lies on the interplay with histone modifications. **(B)** Tyrosine kinases are lacking in yeasts and their activity can be toxic. This allows the investigation of protein variants (in deep mutagenesis screens) that govern kinase activity and function and enables inhibitor testing. Non-toxic low-level expression of human tyrosine kinases in yeast allows large scale screening of phosphorylation dependent protein interaction and the characterization of kinase substrate specificity. **(C)** PARPs attach ADP-ribose to their target proteins. No such modification is reported in yeast. Expression of PARPs in yeast results in a growth phenotype and allows screening for substrates and small molecule inhibitory drugs. **(A-C)** Icons with dark blue background: observable; Icons with light blue background: readout; MS: mass spectrometry; PPI: protein-protein-interaction.

**Figure 2:**
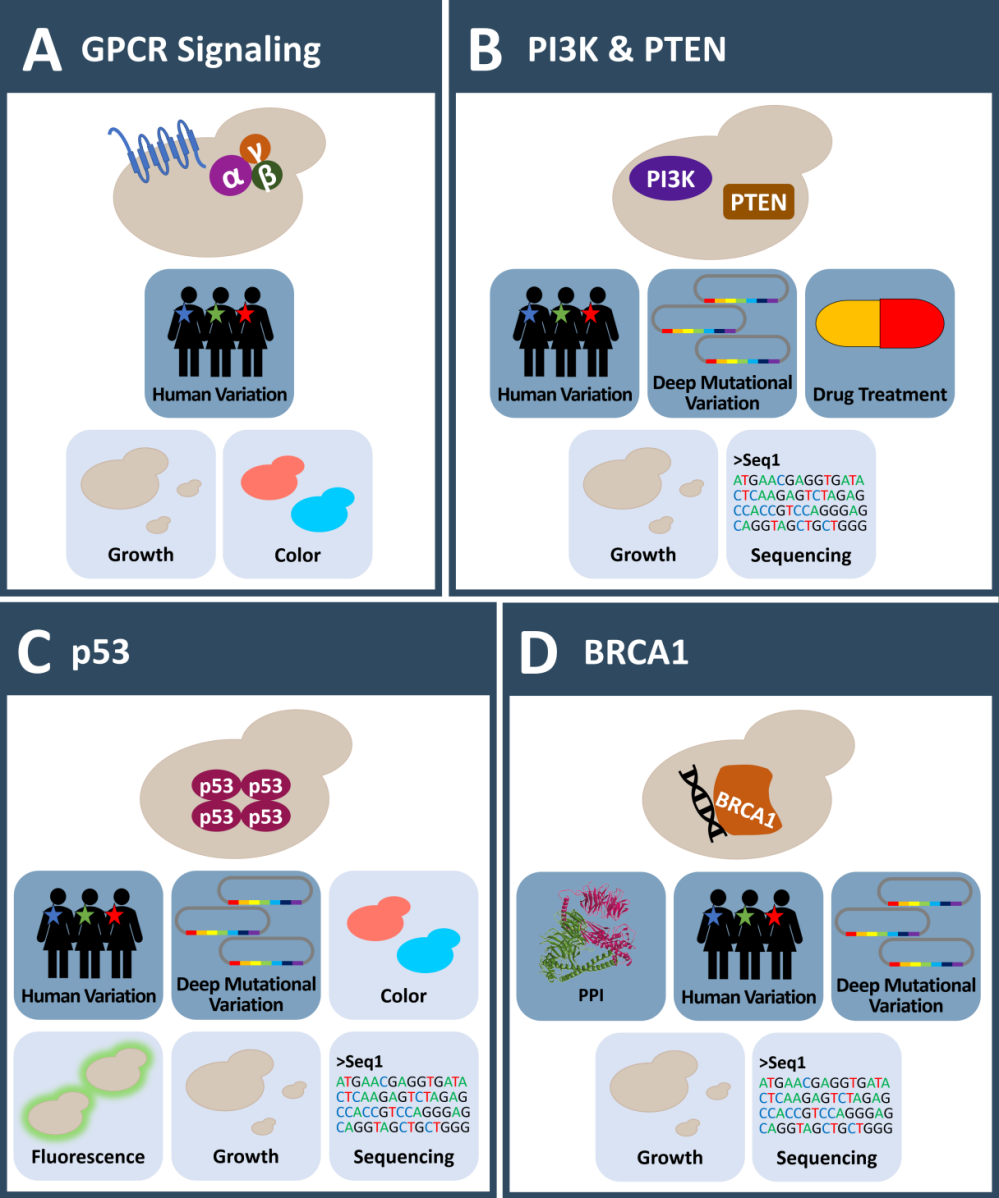
FIGURE 2: Yeast systems to investigate human mutation in cancer proteins. **(A)** GPCR signaling plays an outstanding role in humans, yet only two GPCR related pathways are present in yeast. These pathways can be engineered to investigate human regulators of GPCR signaling and mutations in these proteins in yeast. **(B)** Several aspects of the human PI3K-PTEN-AKT signaling pathway are studied in yeast in the absence of endogenous PI3K activity. Membrane targeting of PI3K in yeast causes a growth phenotype and thus enables the testing of inhibitors. Mutations in PI3K or PTEN frequently found in developing cancers are functionally assessed in yeast. **(C)** Yeast is missing a p53/TP53 homologue. Expression of the human tumor suppressor p53 results in growth defects in yeast. A plethora of assays to investigate the protein function are established in yeast. This allows observing the influence of human variation and the effects of p53 cancer mutations in this system. **(D)** Though *absent* in yeast, expression of the BRCA1 tumor suppressor impacts HR activity of yeast resulting in various phenotypes. This is the basis for the investigation of BRCA1 mutations. Deep mutational scanning of BRCA1 has been applied to comprehensively investigate the effects of amino acid substitutions on protein E3 ligase activity and PPI. **(A-D)** Icons with dark blue background: observable; Icons with light blue background: readout; MS: mass spectrometry; PPI: protein-protein-interaction.

**Figure 3:**
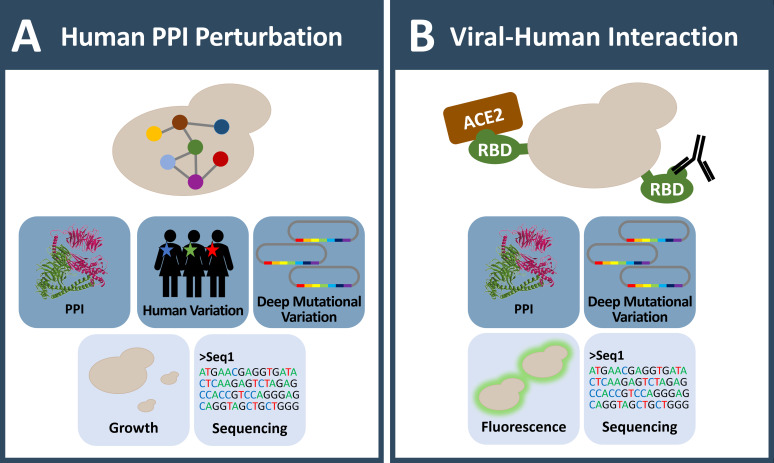
FIGURE 3: Yeast systems involved in large scale protein-protein interaction studies in the context of human coding variation and disease. **(A)** Yeast is used to investigate the influence of human coding variation systematically at large scale. As protein-protein interaction is a uniform functional feature of all proteins, it is especially useful to uncover the influence of variants on protein-protein interaction and consequently its potential impact in disease. **(B)** As a quickly adaptive tool, yeast is currently also used in COVID-19 research. Deep mutagenesis on the SARS-CoV-2 spike protein receptor binding domain (RBD) was performed to characterize mutant variants in human ACE2 receptor and antibody binding. **(A-B)** Icons with dark blue background: observable; Icons with light blue background: readout; MS: mass spectrometry; PPI: protein-protein-interaction.

Exploiting these functional voids creates opportunities where yeast can actually serve as an *in vivo* test tube (**Figure 1, 2 and 3**). In these approaches, human protein functions are grafted on yeast, enabling to study aspects of human molecular functions which are very difficult to assess in the homologous system. While in humans these functions are inextricably linked with multiple aspects and unaccounted regulatory responses of human cell biology, those *absent* functions are isolated in the living yeast. In yeast they can be perturbed free of confounding factors and the effects are direct consequences of the perturbation. They can be read efficiently utilizing phenotypic selection and quantitative functional genomics readouts such as mass spectrometry, DNA sequencing or cell counting/sorting [6]. In other words, the power of assaying *absent* functions in the yeast systems lies in isolating a human activity in a living organism and coupling this activity to yeast based readouts. This approach is fostered by easy genetics, high transformation rates, the possibility to grow a large amount of cell material, parallelization of growth in arrays and a high signal to noise as e.g. growth of a visual colony requires yeast to double 18 times.

Indeed, ectopic, heterologous expression of *absent* functions in yeast very often end in a quantitative growth defect. In the field of neurodegeneration, where overexpression of aggregating disease proteins (e.g. Huntingtin exon 1, alpha-synuclein, TDP-43, Ataxin 2, or amyloid precursor protein (APP)) can cause toxicity, genetic screening was performed to identify yeast genes that suppress the phenotype leading to mechanistic insights, that in exceptional cases could be translated to humans (reviewed in [7–11]). However, mechanistic insight into the toxicity of human protein expression in yeast remains the exception. In most cases molecular mechanisms which lead to the phenotype are elusive.

Irrespective of mechanisms, phenotypic selection enables the detailed analyses of a large number of conditions through enrichment. In particular, testing the function of all possible amino acid variants of a protein in deep mutagenesis screening/scanning approaches is straight forward if non-functional variants can be separated from functional variants through yeast growth selection. Deep mutational scanning approaches follow a “massively parallel paradigm” where all possible single genetic variants of a protein are cloned and subjected to a functional assay and the effects of individual variants are assessed through a sequencing readout [12, 13]. For example, deep mutagenesis scanning of human TDP-43, investigating the relationship between aggregation and toxicity in yeast, revealed that aggregation prone variants are less toxic while toxic amino acid variants promote the formation of liquid-like condensates [14]. A similar deep mutational scanning approach of APP in yeast identified a small set of key residues critically preventing formation of APP aggregates in yeast [15]. With its simple growth phenotypes and growth-based yeast-two hybrid (Y2H), protein complementation or transcriptional reporter assays, yeast has been resuscitated as a powerful tool to study human genetic variation providing most comprehensive functional fitness profiles across all single amino acid mutations of a protein.

Here we discuss selected studies addressing two aspects of systems biology which utilize the concept of *absent* functions in yeast: i) the comprehensive analysis of DNA methylation and post translational protein modifications (PTMs) and ii) the functional assessment of human genetic variation. The latter involves the prioritization of protein variants of frequently mutated cancer genes and application of deep mutational scanning to comprehensively study the effects of coding variation on human protein interactions using yeast systems.

## PROTEIN AND NUCLEIC ACID MODIFICATIONS *ABSENT* IN YEAST

### Targeted cytosine DNA methylation in yeast

In vertebrates, DNA methylation impacts processes such as development, pathogenesis and aging through modulation of gene expression [16]. The modification, which can be detected easily through bisulphite sequencing, occurs at cytosine bases, primarily in the dinucleotide sequence CpG leading to 5-methylcytosine. Increased levels of DNA methylation are, for the most part, associated with decreased gene function. In the context of disease, aberrations in DNA methylation are frequently reported in cancer and used as diagnostic marks. In renal cancer for example, 20% of the tumors show hypermethylation of the tumor suppressor gene VHL [17]. Similarly, the CDKN2A locus is highly frequently silenced through DNA methylation in a variety of cancers [18]. The epigenetic mark is introduced by DNA-methyltransferases (DNMTs). Detailed investigation across various species with a LC-MS/MS approach that achieved subfemto-molar sensitivity confirmed the absence of DNA methylation in *S. cerevisiae, S. pombe* and *Pichia pastoris* [19]. Therefore, baker's yeast can be leveraged to study genomic 5-methylcytosine modification in a background free cellular environment (**Figure 1A**).

To this end, human DNMTs were expressed in *S. cerevisiae* to introduce 5-methylcytosine on the yeast genome [20]. The experiments focused on the interplay between histone modifications and DNA methylation, the latter was measured by HPLC-MS and bisulfite sequencing. Co-expression of DNMT3A and DNMT3L under the control of a *GAL1* promoter resulted in methylation of genomic DNA. However, DNA methylation was abolished by truncation of the N-terminal four amino acids of yeast histone 3 that harbor the H3K4me3 histone methylation mark. Knock-out of *SET1*/*YHR119W, SWD1*/*YAR003W* and *SWD3*/*YBR175W* protein methyltransferases and members of the COMPASS complex, responsible for H3K4 mono-, di-, and trimethylation in yeast, resulted in increased DNA methylation levels. This was underlined by the observation that genomic loci with increased H3K4 methylation clearly had reduced DNA methylation levels in DNMT3 expressing yeast. On the other hand, deletion of the DNMT3L PHD domain or introduction of point mutations disrupting the interaction with histone H3, resulted in loss of DNA methylation [20]. This nicely demonstrated that *de novo* DNA methylation depends on the ability of DNMT3 to bind K4-unmethylated histone H3. Morselli *et al*. recapitulated the effect of H3K4 methylation with human DNMT3B in yeast [21]. Additionally, DNA methylation profiling with whole genome bisulfite sequencing revealed that CpG dinucleotides were favored targets and linker DNA was preferred over nucleosomal DNA. The methylation distribution indicated that within genes methylation was lower at transcription start sites, where H3K4me3 was higher, and increased towards the transcription termination sites, that were enriched for H3K36me. To test if H3K36me3 also influences DNA methylation, H3K36me3 Chip-seq profiles of wild type (WT) and DNMT3B expressing yeast strains were compared. Strong correlation of DNA methylation and H3K36me3 was detected. The use of a knock-out strain of *SET2*/*YJL168C*, responsible for H3K36 methylation in yeast, resulted in reduced DNA methylation levels over gene bodies peaking outside of the gene, indicating that H3K36me3 actively recruits DNMT3B on transcription termination sites. In yeast these studies established that H3K4me3 is strongly anti-correlated while H3K36me3 correlates with accelerated *de novo* DNA methylation [20,21].

A very detailed investigation of human DNMTs was performed in *P. pastoris*, a yeast species also lacking endogenous DNA methylation. Human DNMT1, DNMT3A and DNMT3B were investigated as single knock-ins and in combination with DNMT3L by whole-genome bisulfite sequencing and RNA-seq [22]. Both DNMT3A and DNMT3B produced DNA methylation levels substantially higher than control, additional knock-in of DNMT3L increased the levels one- to three-fold. All combinations again showed the distribution over the gene body and preferred CpG bases. The comprehensive dataset and a time-course experiment in the double knock-in strain expressing DNMT3B-DNMT3L enabled the detection of differentially regulated genes. Downregulated genes were connected to the highest DNA methylation, in agreement with experiments in human cells. As a response to DNMT expression in yeast, RNA-seq initially revealed a large set of differentially expressed genes that monotonically decreased over time (days). Computational analysis attributed the initial peak to stress response. In particular, the availability of S-adenosyl methionine (SAM), an important methyl donor for DNMTs, was negatively regulated, either by reducing the activity of the methionine cycle (decreased expression of *SAM4*/*YPL273W, MET6*/*YER091C*, and *SAM2*/*YDR502C*) or by increased expression of SAM-consuming functions such as enzymes in spermine synthesis or histone methyltransferases. Other transcriptional changes were accounted to generic responses to heterologous expression of human proteins, e.g. upregulation of genes connected to ribosome and protein translation and downregulation of genes connected to mitosis. Overall, methylation state was more predictive for expression decreases compared to expression increases and loci just downstream of the gene TSS were most informative for expression changes.

DNA methylation data were used to train deep neural networks to identify sequence preferences of the DNMTs. DNMT3A and DNMT3B could clearly be separated by local as well as global sequence preferences. Especially methylation by DNMT3A was predictable by local nucleotide sequence features next to CpG nucleotides which were more likely to be methylated together when they were within 30 bp distance. More interestingly, distant CpG nucleotides tended to be frequently methylated together as co-methylation units if nucleosomal DNA wrapping brought distal CpG sites to proximity in 3D space [22].

Yeast as a model for DNA methylation offers valuable insight into *de novo* DNA methylation processes, yet has some limitations. Global methylation rates are much lower compared to mammalian cells. The simple model lacks modification readers that generate sequential effects and known repression marks e.g. on H3K9 or H3K27 or silencing factors reserved to higher organisms (for review on histone modification in yeast see [23]). However, expression of DNMTs in yeast reproduces many aspects observed in mammalian cells, like distribution of DNA methylation in the gene body, influence of histone modifications on methylation levels and diversities among DNMT family members. The simplicity of the *in vivo* test tube model organism overcomes some of the difficulties in the investigation of epigenetics in mammalian cells.

### Assaying tyrosine phosphorylation in yeast

Phospho-tyrosine (pY) signaling is regarded as a hallmark of multi-cellularity and in contrast to serine-threonine phosphorylation, has not evolved in yeast. Orthologs of bona-fide protein tyrosine kinase (PTK) sequences - 58 cell membrane-spanning receptor tyrosine kinases and 32 non-receptor tyrosine kinases (NRTKs) in human - were not found in fungi [24]. As a consequence, there is only a small set of tyrosine phosphorylation sites described in yeast [25] attributed to a few dual-specificity kinases. For example, a tyrosine phosphorylation regulates cell cycle-dependent nuclear localization of Cdc48p, the yeast homolog of VCP [26]. Also, yeast Hsp90 is a target of cell-cycle associated tyrosine phosphorylation by Swe1 kinase, modulating the ability of Hsp90 to chaperone client proteins [27]. However, yeast does not have “true” tyrosine kinases [28, 29] and tyrosine phosphorylation can be regarded as *absent* activity in yeast. Therefore, yeast can serve as an essentially background-free, eukaryotic expression system in which to study tyrosine kinase activity and signaling (**Figure 1B**).

Elevated tyrosine kinase activity in yeast is toxic. Growth inhibition upon overexpression of v-SRC in yeast indicated heterologous PTK activity in the lower eukaryote which later was explained by aberrant phosphorylation of yeast proteins [30–33]. Since yeast growth repression depends on the kinase phosphorylation activity it was used to study function-activity relationships in yeast. The discovery that CSK induces SRC autorepression by C-terminal tyrosine 530 phosphorylation of SRC stems from using a heterologous yeast system, where SRC expression induced toxicity could be rescued by co-expressing human CSK [34]. Several pY-sites on FYN kinase were characterized in yeast as autophosphorylation sites [35]. Activity and autoregulatory mechanisms of various tyrosine kinases such as SRC, ABL, FES, and HCK were investigated in yeast, exploiting the absence of inhibitory factors [36–40].

The toxic effect of tyrosine kinase activity in yeast can be leveraged to study kinase activity perturbations, and as such was exploited for screening of inhibitors of kinase or phosphatases which restore yeast growth [41–43]. Similar to the co-expression of repressive CSK kinase in the presence of SRC [34], co-expression of the catalytical domain of PTP1B tyrosine phosphatase can rescue v-SRC tyrosine kinase growth phenotype. This system was utilized as a cell-based assay, where PTP1B dependent growth served as a biological read out for the effect of PTP1B inhibitors such as vanadate, BzN-EJJ-amide, Suramin, Phenylarsine oxide and other non-peptide PTP1 inhibitors [41].

Recently, Ahler *et al*. used a deep mutational scanning approach to dissect intramolecular regulation of SRC NRTK [44]. SRC kinase activity-dependent decrease in yeast growth allowed to select protein variants from a pool of single amino acid substitution in the catalytic domain of SRC covering about 70 % of all possible single mutants. Competitive growth selection identified 46 residues where over 90 % of the substitutions resulted in loss of function. However, 27 residues in the catalytic domain had at least five gain-of-function mutations, i.e. were depleted in the pool. For example, every substitution of D368 was activating, as this residue contributes to the autoinhibitory interaction with the SH2 domain. Also known SH3 domain intramolecular inhibitory interactions involving SRC residues 289-293 were mapped well. Further analyzes of the deep mutagenesis results clustering around residue E381T in different mammalian cell lines revealed an unknown function of the N-terminal SH4 domain. It interacts with the αF pocket of the C-lobe in the catalytic domain and thereby enhances the known autoinhibition effect of the SH2/SH3 domains [44].

Toxicity of tyrosine phosphorylation was reported as a consequence of the expression of many different human tyrosine kinases, which indeed showed very different, i.e. specific, phosphorylation patterns in yeast, when the yeast proteome was probed with pan anti-tyrosine antibody [45]. Together with the fact that growth defects correlate with the overall level of tyrosine kinase activity, this suggested a more general mechanism that led to toxicity. One proposal was that the lethality of v-SRC should be attributed to the hyper-Y-phosphorylation of multiple yeast proteins leading to mitotic dysfunction but the exact target(s) responsible for mitotic dysfunction were not identified [41]. Recently it was reported that overexpression of yeast Smk1p (YPR054W), a MAP kinase required for production of the outer spore wall layers, alleviates toxicity induced through *GAL10* promotor controlled expression of the human SRC protein [46]. Whether Smk1p overexpression in general rescues pY related toxicity was not tested, both the mechanism of toxicity as well as mechanism of growth rescue through Smk1p expression remain elusive.

However, while high tyrosine kinase activity clearly is growth inhibitory, yeast tolerates tyrosine phosphorylation to quite some extent (as measured by tyrosine phosphorylation of the yeast proteins), opening other routes for assaying this *absent* modification in a yeast *in vivo* model system. A systematic use of low level NRTK co-expression in yeast enabled screening of pY dependent human protein interactions [47]. Screening 188 human open reading frames (ORFs) in the presence of nine tyrosine kinases (each as a representative of a different NRTK family) against a human proteome scale matrix identified ~ 300 novel pY-dependent protein-protein interactions (PPIs) that showed high specificity with respect to kinases and interacting proteins. Although this approach did not directly reveal which tyrosine site is responsible for the interaction, it unambiguously established the phosphorylation dependent nature of the interaction. The data support the notion that many of the pY-interactions are not mediated by linear recognition motifs encouraging the investigation of novel modes for pY modification recognition. The utility of yeast as Y-phosphorylation free system was highlighted by the fact that the PPIs were shown to be selectively catalyzed through distinct sets of NRTKs. In contrast, phosphorylation requirements were masked in fast growing mammalian cell lines due to high intrinsic tyrosine kinase activity [47].

In a complementary approach, low level human NRTK expression in yeast was used to study human tyrosine kinase phosphorylation specificity [45]. To this end, active full-length human PTKs were expressed and the yeast proteome was analyzed through mass spectrometry based pY-proteomics. In this approach the proteome of the growing yeast cell served as *in vivo* model substrate for an individual human kinase, which was acting in an intact, crowded, cellular environment. Every tyrosine phospho-site could be unambiguously attributed to one human kinase. Assaying 16 of the 32 human NRTKs revealed ~ 3700 kinase-substrate relationships. *De novo* linear kinase motifs derived from yeast-phospho-sites for all 16 kinases showed predictive performance equal or better than known motifs [48–50]. However, the key finding came from analyzing the phospho-data in the context of yeast PPI networks [45]. It demonstrated that human tyrosine kinases preferentially target interaction network structures in yeast, a living organism that did not evolve *bona fide* tyrosine signaling. The average number of interacting pairs of phosphorylated yeast proteins in the yeast protein interaction network was much higher than expected. Similarly, the average shortest path between pairs of phosphorylated yeast proteins for a given kinase was significantly smaller than in corresponding randomized networks. This very strong signal of network connectivity demonstrated that substrates of an individual kinase cluster in the protein interaction network. Hardly any significance was observed in functional gene ontology (GO) enrichment analysis. Hence, the observed network signature is unrelated to any phosphorylation driven function. The data were the first direct experimental evidence supporting the hypothesis that tyrosine kinase-substrate specificity determinants are at least in part governed by PPIs in cellular networks.

Kinase perturbation studies in yeast [51, 52] and mammalian systems [53–57] demonstrate that kinases form complex signaling networks including redundancy and feedback loops [58, 59]. While altered pathway signatures and kinase regulation profiles can be inferred from phospho-proteomics data of perturbed systems [60], the assignment of direct kinase substrate relationships remains for the major part elusive. Cell biological, biochemical and bioinformatic approaches have only identified kinases for less than 5% of the phospho-proteome [61], which includes about 50,000 pY sites in human. As each kinase on average has more than 500 substrates, the numbers also challenge the classical key-lock principle of kinase (enzyme)-substrate recognition [62, 63]. The demonstration that tyrosine kinase-substrate specificity is governed by protein interaction networks [45] provides a first clue to the question of how the relatively small number of < 100 tyrosine kinases can address a major part of the proteome modifying 50,000 non-redundant sites. A network driven phosphorylation specificity model would allow the kinase to modify essentially all available tyrosine sites on a large number of substrates. Fostered through protein interaction, protein tyrosine residues that spatially and temporally reach the active site of the kinases will be phosphorylated – without specific recognition requirements. This hypothesis is in agreement with a crystal structure of the EGFR kinase domain bound to an optimized substrate peptide in the active center that showed no defined electron density for the substrate side chain residues. Begley *et al*. concluded that, other than the +1 residue, the primary sequence surrounding the phosphorylation site may have little influence on EGFR kinase specificity [64]. The network driven tyrosine phosphorylation model is also in agreement with the lack of any linear sequence motif signature for more than 80% of recorded human phospho-sites and the observation that cellular changes are typically accompanied by broad quantitative alterations of the phospho-proteome [65–68].

### Introducing poly(ADP-ribosyl)ation to yeast

Poly(ADP-ribosyl)ation is a post-translational protein modification (on D, E, and/or K side chains) which controls cellular processes such as DNA repair, transcriptional regulation, cell division, protein degradation, apoptosis and necrosis. It has a key role in response to cell stress and DNA damage [69]. Poly(ADP-ribose) polymerases (PARPs) transfer ADP-ribose from NAD+ to target proteins forming long linear or, less frequently, branched chains [70, 71]. Among all functional features of PARP1, the most famous is probably the synthetic lethal genetic interaction with BRCA1. In the clinics PARP1 inhibitors are successfully used to treat BRCA1 mutated cancers [72, 73]. However, many aspects of the broad variety of PARP cellular functions are not yet fully understood. PARPs are present in many higher organisms and bacteria, yet are *absent* in yeasts [74].

Expression of full-length human PARP1 in *S. cerevisiae* results in a low number of yeast colonies, with yeast cells of elongated shape and increased size (**Figure 1C**). The DNA binding motif in the N-terminus is important for the growth suppressive effect. A truncated version of PARP1, lacking this domain, or *Arabidopsis thaliana* PARP isoforms without DNA binding motive sustained yeast growth [75,76]. Cell cycle analysis demonstrated a G2/M block [77]. Yeast cells that expressed PARP1 responded to the DNA damaging agent N-methyl-N'-nitro-N-nitrosoguanidine (MNNG) with increasing PARylation levels in a dose-dependent manner. However, PARP1 expression reduced cell viability when additional factors like radio-labelling or MNNG treatment were combined. Surprisingly, levels of NAD+ were not altered when PARylation was detected. This discrepancy might be explained by the large pool of NAD+ present in yeast [78]. Addition of NAD+-binding inhibitors like 3-methoxybenzamide and other PARP1 inhibitors like 6(5H)-phenanthridinone (PHE) to the growth medium abolished the growth suppressive phenotype, which suggests the enzymatic activity of PARP1 being required for the effect [75, 79]. A drug screen exploited the ability of PARP1 inhibitors to suppress the growth defect of yeast strains overexpressing PARP1 or PARP2. From the 16,000 small organic compounds contained in the drug library, ten new compounds restoring growth were identified [79].

Direct identification of PARP1 interacting yeast proteins with a yeast proteome microarray assay revealed 33 PARylated yeast proteins. Further validation with an autoradiography assay, measuring incorporation of radiolabeled NAD+, showed PARylation on PARP1 itself as well as three yeast proteins (Nob1, Has1 and Lhp1). *In vitro* PARylation experiments with the human homologues confirmed the finding from yeast and suggested a role for PARP1 in ribosome biogenesis [77].

In addition to yeast growth inhibition, LaFerla *et al*. [80] found that expression of PARP1 reduces inter-chromosomal recombination after UV induced DNA-damage. The PARP1 inhibitor PHE reversed the effects. To find direct interacting yeast proteins responsible for the observations, a library of yeast knock-out strains was screened for rapid growth in the presence of PARP1. Ninety-nine deletion strains were collected, and subsets were validated individually in growth assays. Since reduced chromosomal recombination after UV treatment and growth inhibition upon PARP1 expression occur together, the identified yeast proteins were investigated on their ability to change PARP1-GFP localization after UV treatment. *HHO1*/*YPL127C* and *POM152*/*YMR129W* knock-out strains increased the nuclear localization of PARP1. The results raised the hypothesis that the homologous genes might also alter PARP1 localization in human [80]. Only recently, the dataset obtained from this yeast screen was re-analyzed computationally. Since twelve target genes were associated with neoplasms, data from eight cancer types and over 2,000 patients were scrutinized for co-occurrence or mutual exclusivity of gene amplification, deletion or mutation with PARP1. In prostate cancer nine of the twelve target genes showed an expression correlation especially striking with INCENP. Another expression correlation was found with PARP1 inhibitor treatable triple-negative breast cancers and higher PSAT1 and RIT1 expression compared to non-treatable hormone receptor breast cancers. The finding was tested by small interfering RNA (siRNA) induced silencing PSAT1, RIT1 or INCENP in MCF7 breast cancer cells and treatment with the PARP-inhibitor Olaparib. Knock-down of PSAT1 and INCENP rendered the cells more sensitive to the PARP1 inhibitor pointing towards a novel drug target combination to increase PARP1-inhibitor efficiency [81].

To summarize, expression of PARP1 in *S. cerevisiae* results in reduced growth and PARylation levels can be monitored easily. PARylation depends on the protein's enzymatic activity and is responsive to DNA damage. In contrast to mammalian cells the amount of NAD+ as substrate for PARylation appears not limiting in yeast, which might influence the outcome and interpretation of PARP1 activities. However, yeast has successfully been utilized as *in vivo* model of Poly(ADP-ribosyl)ation in small molecule drug testing, interaction and substrate profiling. The system could also be used to investigate mutants of PARP, exploiting PARylation activity through defined yeast growth phenotypes.

## HUMAN VARIATION OF CANCER PROTEIN FUNCTIONS *ABSENT* IN YEAST

### GPCR signaling in yeast – Regulators of G protein signaling (RGS)

GPCR (G protein coupled receptor) pathways play important roles in the processes of homeostasis and especially in cell signaling. These functions are often dysregulated in disease and recent studies show that currently around 35% of FDA-approved drugs target GPCRs (reviewed by [82]). There are several hundreds of GPCRs in human, whereas *S. cerevisiae* holds two important GPCR pathways only, the glucose sensing pathway (*GPR1*/*YDL035C*) and the pheromone responsive pathway (*STE2*/*YFL026W*; *STE3*/*YKL178C*). The latter is the homologue of the human MAPK pathway and necessary for the yeast mating response. With its simplicity, yeast allowed the first description of an α-factor pheromone dependent G1-cell cycle arrest linked to mutations in Sst1 (YIL015W) or Sst2 (YLR452C) [83, 84]. The following connection to the negative regulation of G-protein signaling [85] and the detailed mechanism of the Sst2-dependent activation of Gpa1-GTPase activity [86] was also discovered in yeast.

Experimental setups to investigate human GPCRs and human Gα subunits (GNAs) by exchanging yeast to human homologues exist in large variety (review on GPCR signaling in yeast by [87]). Knock-out of the yeast GPCRs uncouples it from hormone response. Expression of a chimeric Gα-subunit and fusion of the downstream transcriptional *FUS1*-promoter to a *HIS3*- or a *LacZ*-reporter renders the system capable to identify human regulators of G-protein signaling (RGS proteins) by expressing human cDNAs in yeast (**Figure 2A**). In this system, Gα-dependent activation of the pathway was achieved with AGS1 expression and the protein family of activators or G-protein signaling were first described [88]. AGS1 acts as a guanine nucleotide exchange factor and interacts directly with Gαi2. RGS5, a GTPase-activating protein for the Gαi proteins, was identified as a negative regulator in this yeast experiment [88]. Later, AGS1 was also found to interact directly with the Gβ1 subunit in a Y2H screen [89].

In one recent example of using a yeast system to exploit human RGS proteins, 49 sequenced disease variants of 17 RGS proteins were expressed in yeast and the effect on their GTPase activity was investigated [90]. The yeast strain carried a knock-out of the endogenous GPCR *STE3*/*YKL178C*, contained a chimeric Gα-subunit, had the downstream *FUS1* promoter fused to a *LacZ*-reporter for a readout and lacked *FAR1*/*YJL157C* to prevent cell cycle arrest. AGS1 was co-expressed for constitutive activation of the pathway. The GTPase-activating protein (GAP) activity of WT and mutant RGS was compared to the expression of activating AGS1 alone using a β-galactosidase assay. Results show that mutations located on the surface of the proteins were more likely to have a severe functional GAP activity defect. Nine mutants were further validated in a BRET assay in mammalian cells recapitulating the observed effects from yeast [90].

This recent experiment addressing disease-related amino acid variation of RGS proteins is just one of many applications for the investigation of human GPCR signaling in yeast. The presence of only two GPCR pathways in *S. cerevisiae* compared to hundreds in human simplifies investigation. Considering that GPCRs are the major class of drug targets, and that the downstream pathway members are often mutated in human disease, investigation of disease variants in yeast could rapidly identify causal mutations and lead to novel approaches for intervention.

### PI3K/PTEN function in yeast

Class 1 phosphoinositide3-kinase (PI3K) is composed of two subunits, the p110 subunit, required for the catalytic activity and one of the p85/p55 regulatory subunits. In mammalian cells, the kinase activity is required for phosphoinositide signaling. Upon stimulation at the plasma membrane, the human class I PI3K converts phosphatidylinositol 4,5-bisphosphate (PIP_2_) into the second messenger phosphatidylinositol (3,4,5)-trisphosphate (PIP_3_). The synthesized PIP_3_ leads to the activation of AKT kinase, which phosphorylates proteins involved in cell growth and cell survival. This signaling pathway can be turned off by the phosphatase PTEN, which catalyzes the conversion of PIP_3_ to PIP_2_ [91]. Perturbed regulation of this pathway due to sustained AKT signaling or loss of PTEN function can promote cancer development and progression and diabetes [92, 93]. In certain cancers such as breast cancer, virtually all nonsense substitutions and over 90% of missense substitutions in PTEN are driver mutations [94].

*S. cerevisiae* lacks a class I PI3K homolog which allowed the study of the human PI3K-PTEN-AKT signaling pathway in the absence of endogenous PI3K activity (**Figure 2B**) [95, 96]. Expression of the human catalytic PI3K p110 subunit, fused to sequences targeting the kinase to the yeast membrane (e.g. a CAAX-box) or as a cytosolic version in combination with a regulatory p85 subunit fused to a CAAX-box, strongly decreases yeast growth [92, 97]. This growth phenotype is related to the depletion of the membrane PIP_2_ pool, causing altered morphogenesis and vesicle trafficking [92]. Co-expression of PI3K and human AKT, leads to AKT activation and ultimately to a direct activation of the TORC2 pathway, skipping endogenous signaling pathways [98].

Baker's yeast can be used as model organism to assess the potency of PI3K inhibitors, highly relevant for cancer treatment. In these assays, the catalytic PI3K p110 subunit was expressed in yeast and following inhibitor treatment growth rates were used to determine the ability of the drug to inhibit PI3K [99, 100]. Evaluation of the screening hits in other cell-based assays, including mammalian cells, confirmed the results obtained in the yeast assay, highlighting the importance of these assays as primary drug screening platforms [99].

Tep1/YNL128W is an inactive yeast homolog of PTEN, with no demonstrated inositol lipid phosphatase activity and thus unable to counteract the human PI3K catalytical p110 subunit. In contrast, co-expression of active human PTEN, including two less characterized N-terminal splice variants (PTEN-L and PTEN-M), robustly rescues the observed growth phenotype, comparable to the treatment of yeast cells with PI3K inhibitors [92, 101]. PTEN mutations are associated with cancer development and PTEN hamartoma tumor syndrome, but also patients with autism spectrum disorder carry PTEN mutations that can be characterized exploiting the yeast phenotype [102]. Recently, 141 PTEN cancer nonsense mutations were classified as WT like, reduced or inactive variants using G418 as readthrough-inducing compound in human cells and in the yeast heterologous system [103].

Mighell *et al*. [104] studied PTEN mutants and their effect on the phosphatase activity. A deep mutagenesis library harboring 95% of all possible PTEN mutants was generated and introduced in a yeast strain co-expressing PI3K fused to a prenylation box motif. Evaluating growth as a readout of relative phosphatase activity, a fitness score was calculated for each variant. 2,273 (31%) of the tested mutants were classified as likely damaging whereas 4,872 (67%) amino acid variants were classified as WT-like. Comparison of deep mutagenesis results with the crystal structure of PTEN showed that mutations located in the phosphatase domain close to the catalytic pocket were deleterious, especially Arg130 did not tolerate any amino acid substitution, while mutations in unstructured regions were quite flexible with respect to single amino acid mutations [104]. Reviewing and combining results of deep mutagenesis approaches which used distinct readouts - in yeast and mammalian systems - revealed a small set of key variants that specifically abrogate distinct molecular functions of PTEN and provided a functional classification of pathogenic variants [105].

### The p53 tumor suppressor protein in yeast

The tumor suppressor p53 is one of the most frequent mutated proteins in cancer, and also one of the most intensively studied proteins. *S. cerevisiae* lacks a p53 homolog and expression of the human protein results in a decreased growth rate [43, 106–109]. Nigro *et al*. showed that the expression of p53 in yeast increased the doubling time up to 250% compared to empty vector control or the expression of an inactive mutant version of p53 [108]. The ability to express active p53 in a background free model organism made yeast-based assays a common tool to study the relevance of a p53 mutation on its ability to act as a tumor suppressor (**Figure 2C**).

In 1990 Fields and Jang [110] showed that p53 can activate transcription in yeast. Using a yeast strain with an integrated *GAL4-LacZ* reporter gene, they demonstrated that the *ADH1*-promoter driven expression of a Gal4-p53 hybrid protein could activate transcription leading to β-galactosidase activity. Furthermore, they demonstrated that high level expression of human p53 is detrimental to the yeast growth and phenocopies the effects in mammalian cells [110].

Schärer and Iggo [111] demonstrated that mammalian p53 on its own can act as a transcription activator in *S. cerevisiae*. They implemented an assay that allows the expression of *LacZ* under control of a *CYC1*-hybrid promotor, containing a p53 binding site. While WT p53 was capable of binding to the promotor and thereby leading to the transcription of *LacZ*, they could demonstrate that three known mutations associated with the Li-Fraumeni syndrome (tumorigenesis) failed to bind to the promoter and therefore were inactive [111]. This experiment formed the basis for FASAY, Functional Analysis of Separated Alleles of p53 in Yeast, which is used since then to detect non-functional p53 mutants derived from patient samples [112].

For FASAY, mRNA of the patient sample is extracted, amplified via RT-PCR and transcribed into cDNA, which is then introduced in a yeast strain and cloned into a plasmid via gap repair. First generation FASAY [113, 114] used the ability of p53 to activate transcription via a *HIS3*-based reporter system with growth as a phenotypic readout. Functional p53 mutants maintained yeast growth, while non-functional p53 mutants failed to activate *HIS3* transcription and hence did not grow in the absence of histidine [114]. Flaman *et al*. [115] introduced the colorimetric FASAY, which uses a red colony phenotype to detect missense p53 mutants. The p53 promoter binding site is fused to *ADE2*, and adenine deficiency accumulatively leads to red colored yeast in this genetic setup. Hence, functional p53 mutants result in white colonies while colonies with non-functional p53 mutant proteins turn red. Furthermore, this assay allows to gradually distinguish partially active p53 mutants (pink colonies) from completely active and inactive mutants [112, 115]. Jia *et al*. demonstrated that the FASAY can be used to determine the p53 status of 142 tumor cell lines. More than 70% showed mostly homozygous mutations in the TP53 gene, and thus are a common feature in immortalized cancer cell lines [116].

FASAY underwent ongoing improvement enabling the measurement of p53 mutant activities on other promotors. Flaman *et al*. [117] developed an assay version, which allowed to detect mutants that specifically affect transcriptional activity of p53 on the p21/CDKN1A and BAX genes. Activation of p21 leads to a G1 arrest while BAX transactivation leads to apoptosis. Due to a mismatch in the DNA promoter sequence, the p53 interaction with the BAX promoter has a lower affinity compared to the p21/CDKN1A responsive elements, and thus mutations are more likely to impair BAX promoter binding. Screening for the red/white yeast phenotype using an *ADE2* reporter revealed that tumor-derived p53 mutants were able to transactivate p21/CDKN1A, yet failed with BAX [117].

To assess the dominant negative potential of a p53 mutant through tetramerization, FASAY was adjusted to allow the expression of two different p53 alleles (e.g. mutant and WT) in a yeast cell. The overall activity of the two variants, expressed from two identical vectors that only differ in the selection marker sequence, is measured as ability to activate an *ADE2* reporter under the control of a p53-responsive promoter. Again, white-colored colonies represent a recessive mutant and dominant p53 variants show red or pink colonies, accounting for the effect of a mutant on the p53 tetramer in heterozygous cells [118, 119]. Monti *et al*. [120,121] used the adjusted FASAY to assess the potential of p53 mutants to have a dominant negative function. Out of 71 tested p53 mutants, 20 acted as dominant negative over WT in their ability to bind the *RGC* responsive element [120]. Their results generally indicated, that the amino acid position of the mutation governs the dominant negative effect, irrespective of the identity of the amino acid exchange. Their study also revealed a hierarchy of transdominance on promoters containing three different p53 responsive elements (such as the p21/CDKN1A, BAX or PIG3 p53 binding sites), consistent with the idea that a weaker interaction between p53 and the responsive DNA element is more prone to be affected by p53 mutations. Billant *et al*. [112] used the FASAY to further investigate the dominant-negative effect of some p53 mutants. In total seven hotspot p53 mutants and 24 isoforms of p53 and its related genes p63/TP63 and p73/TP73 were tested. Their study showed that only p53 mutants that were transcriptionally inactive but still capable to form tetramers could exert a dominant-negative effect over WT p53, most likely by tetramer poisoning. Furthermore, this dominant-negative interference was not limited to p53 and also involved p63 and p73 isoforms. These results highlight the broad applicability of the FASAY as its use to determine potential crosstalk between the isoforms of p53 and its related proteins p63 and p73 [122]. The above description of the FASAY is just a small snapshot from a large variety of available assay versions, highlighting the importance and diversity of yeast-based assays in the field of p53 research. Indeed, the use of the FASAY has been extensively reviewed [112, 123, 124]. In addition to FASAY, there are other yeast-based methods that determine the effect of a specific mutation on p53 function, e.g. Andreotti *et al*. implemented a dual luciferase system to screen for p53 activity in yeast [125].

The first comprehensive mutational analysis on p53 missense variation in yeast included all 2314 p53 single point mutations that can be obtained from one base mismatch throughout the full-length protein [126]. Kato *et al*. used a PCR-mediated mega primer method to introduce mutations in combination with gap repair to insert the mutant p53 versions in the plasmids directly in yeast. EGFP expression was under the control of p21/CDKN1A-p53 binding element, and the resulting green fluorescence was used to identify p53 mutants that could bind the p21/CDKN1A promotor. By mating the array of p53 mutant expressing strains with a strain that carried a red fluorescent protein (DsRed) under control of the MDM2 promotor p53 binding element, the same mutant could be tested simultaneously for its effect on two DNA binding sites. In their set of mutants, they observed that mutations in the DNA-binding site of p53 decreased the fluorescence signal. 55.2% of the p53 single amino acid variants retained activity for both promoters and 17.3% were totally inactive. However, 27.5% of the mutants lead to a differential reduction of reporter activities, supporting the hypothesis that sequence-specific activation might be critical for the tumor suppressive function of p53 [126]. This study is a very early prime example of the power of deep mutational scanning approaches to study human protein variation in yeast.

### BRCA1 activity in yeast

Breast cancer type 1 susceptibility protein, encoded by the BRCA1 gene, is an 1863 aa long protein acting as a tumor suppressor which is involved in many different cellular processes like DNA repair mechanisms, DNA damage response and transcriptional regulation [127, 128]. Mutations in this gene strongly predispose to ovarian and breast cancer and germline mutations lead to a high prevalence of inherited breast and ovarian cancer within families [127]. *S. cerevisiae* is lacking a BRCA1 homolog and expression of human BRCA1 leads to decreased growth, as evidenced by a small colony phenotype [129]. Several assays using *S. cerevisiae*, based on different cellular mechanisms were developed to assay BRCA1 missense variants (**Figure 2D**). These assays include the small colony phenotype assay (SCP) [130, 131], a localization assay [132], a protein interaction assay [133], a homologous recombination (HR) assay [134] as well as a transcription assay [135]. The benefits and limitations of these yeast assays have already been extensively reviewed [124] including a recent review focusing more broadly on human DNA repair genes [136].

Many DNA repair mechanisms are conserved from yeast to human, which makes *S. cerevisiae* a suitable model organism to study BRCA1 variants and their effects on HR [136–139]. Despite the lack of BRCA1 in yeast, the HR assay, implemented by Caligo *et al*., measures the ability of BRCA1 variants to affect spontaneous HR in yeast. A yeast strain carrying two truncated *HIS3* alleles is used. While one allele has a truncated 3' end, the other has a truncated 5' end and 400 bp are shared. Expression of known cancer related missense mutant BRCA1 (e.g. A1789T or M1775R) leads to an increased HR and thereby the frequency of recombination of the two truncated *HIS3* alleles is increased compared to WT. This results in an increased number of viable cells on agar lacking histidine [134]. Maresca *et al*. used the HR assay to investigate the interplay between human MSH2, a protein that recognizes DNA mismatches, and BRCA1 in yeast. Their data suggest that a functional relation between these two proteins may also exist in yeast and that this interplay is required for HR [128]. Using the yeast HR assay, Lodovichi *et al*. looked into the effects of recombination after methyl methane-sulfonate (MMS)-induced DNA strand breaks. Yeast expressing WT BRCA1 or known classified BRCA1 mutants was treated with the alkylating agent MMS and the effect on recombination was measured by cell survival. Cells expressing BRCA1 cancer variants were more sensitive to MMS and less prone to recombination as compared to cells expressing WT BRCA1. Thus, cancer associated BRCA1 missense variants C61G and M1775R showed a decreased recombination after MMS-treatment providing further evidence that human BRCA1 can interfere with yeast DNA repair [140]. The interplay between BRCA1 and other enzymes, besides MSH2, in yeast HR was investigated by Maresca *et al*., who generated strains lacking *MRE11*/*YMR224C, RAD50*/*YNL250W, RAD51*/*YER095W* or *MSH6*/*YDR097C*, all crucial for yeast HR, and performed the HR assay with WT BRCA1 and mutants. While BRCA1 mutants were capable of increasing intra-chromosomal recombination in the HR-strain, they failed to do so when one of the above-mentioned enzymes was lacking. This suggests that human BRCA1-mediated genome instability can only be introduced when the endogenous yeast DNA repair machinery is intact [137]. All these studies clearly demonstrate that, although BRCA1 is *absent* in *S. cerevisiae,* it represents a useful model organism to study BRCA1 and its effects on HR [136].

Another important cellular function of BRCA1 is its interaction with BARD1 via the RING domain and the resulting E3 ubiquitin ligase activity. Testing BRCA1 mutants in a Y2H as well as a yeast split-hybrid PPI assay, the N- and C-terminal helices flanking the RING domain of BRCA1 were found to be involved in the interaction with BARD1 [141]. The study was extended to test BRCA1 mutants on their ability to interact with BARD1 and UbcH5a/UBE2D1, the E3 and E2 ubiquitin conjugating enzymes, respectively. A library generated by random mutagenic PCR containing N-terminal BRCA1 RING mutants was tested in a yeast split hybrid assay, identifying residues that were required for the interactions with E2 or BARD1. Thirty-five BRCA1 mutations also described in patients were retested for the ability of the BRCA1-BARD1 complex to interact with UbcH5a/UBE2D1 and thereby to form ubiquitin chains. In accordance with the yeast split hybrid results, only mutations located in the Zn^2+^-ligating residues disrupted the activity of the ubiquitin ligase [133]. The interaction approach to study BRCA1 mutations was taken further to massive parallel mutational scanning screens. Starita *et al*. [142] assessed the effects of approximately 2,000 missense mutations of BRCA1 on the interaction with BARD1 and on its homology-directed DNA repair activity. To this end, the effects of the variants on heterodimerization with BARD1 RING domain and the E3 ubiquitin ligase activity were tested in two independent yeast and mammalian functional assays, respectively. A multiplexed Y2H assay was used to test the ability of the generated mutants to interact with the BARD1 RING domain, obtaining BARD1-binding scores for the individual mutations. Overall, these scores agreed with the knowledge about the RING-RING interaction, although primarily residues coordinating the zinc fingers scored highest. Combining both data sets, a prediction tool was developed classifying patient mutations as pathogenic or benign. This study nicely demonstrates the power of yeast-based assays in the context of massive parallel screening and deep mutagenesis approaches.

## UP FOR SYSTEMATICALLY TESTING IN YEAST: CODING VARIATION SUBSTANTIALLY MODULATES PROTEIN-PROTEIN INTERACTION

### Impact of amino acid mutation on human protein interaction

Each individual human genome carries tens of thousands of non-synonymous coding variants including 50-200 excess rare deleterious changes [143,144]. Many more coding variants are observed in cancer genomes [94]. Whether or not a variant and combinations thereof have any biological or even physiological effect(s) remains undetermined for the majority. Yeast can be used in functional complementation assays to assess the impact of variants from human disease genes [145]. In addition, evidence that the phenotypic effects of amino acid variation substantially relate through changes of protein interaction patterns is growing. Alterations of amino acid identity at PPI surfaces can have deleterious consequences and underlie altered genotype to phenotype relationships in particular in human genetic diseases [146,147]. The extent to which natural human population variation or disease associated variation, as determined from cancer genome sequencing, modulates protein interaction patterns is still elusive [148–152]. Powerful protein interaction detection methods such as dihydrofolate reductase protein complementation or the two-hybrid system in yeast enable the individual testing of hundreds of coding variants [150, 153, 154]. Moreover, these techniques were further adapted to deep mutagenesis approaches that enable to chart mutational effects on protein-protein interactions comprehensively [147].

First systematic studies to test the effects of single amino acid substitutions on protein interaction behavior were performed using the Y2H system (**Figure 2D and 3A**). An initial study of 29 allelic disease variants which were associated with five distinct genetic disorders [146] revealed that in 16 instances the interaction profile of the protein was changed while maintaining its function in the assay. The concept that disease associated amino acid substitutions alter protein interactions rather than a protein function as such was nicely illustrated in the case of the Wiskott-Aldrich syndrome protein (WASP) harboring rare disease mutations in its protein interaction surfaces. While mutations in the PBD domain [I294T] disrupt interactions with CDC42, mutations in the WH1 domain [R41G] disrupt interactions with TRIP10 and ABI3. The former mutation is causing X-linked neutropenia while the latter mutations are linked to Wiskott-Aldrich syndrome and X-linked thrombocytopenia [149, 155]. A second example, different mutations in TP63 observed in at least two clinically distinct types of developmental ectodermal dysplasia, can be rationalized through interaction perturbation. The causal mutations are in two separate domains, one in the p53 DNA-binding domain and the other in the SAM2 domain mediating a PPI with TP73 [146, 149]. These studies provide conceptionally strong examples on how coding variation translates through interaction changes into phenotypes. Many more interaction modulating disease protein variants were predicted through computational modeling approaches of protein interaction interfaces [148, 149, 151, 152, 156], yet the extent to which interaction perturbation caused by coding variation governs human disease is less clear [157].

To address this question, Wei *et al*. [153] assayed 204 mutations in 51 human proteins in yeast and classified mutations in three groups: i) interface residues; ii) interface-domain mutations and iii) away-from-the-interface mutations. After excluding allelic variants that showed altered protein stability 72% (13/18), 51% (42/83) and 17% (9/52) of the mutations disrupted interactions in the three groups, respectively. In the context of a large Y2H approach to measure interaction perturbations in human genetic disorders, fine dissection of interaction patterns involving 197 mutations in 89 proteins with two or more interaction partners revealed that 31% of the mutations (in 39 proteins) led to selective loss of protein interaction partners [154]. These studies demonstrate that a high fraction of disease-associated amino acid substitutions impact protein interactions. The systematic study of amino acid mutations that perturb protein interactions is not restricted to the analysis of annotated disease variation. Indeed, Fragoza *et al*. [150] evenly sampled population, somatic, and disease-associated variants with amenable PPIs. Importantly, the ~ 2,000 population variants (taken form the ExAC repository) were representative of different allele frequency ranges from rare to common. When testing mutant proteins against multiple interaction partners in Y2H, they found that about 16% of population variations were selectively disruptive for protein interaction while disruptive population variants seldom resulted in unstable protein expression. The fraction of disruptive variants decreased inversely with increasing allele frequency. While on average more than 20% of the rare mutations (<0.1% frequency) did disrupt interactions, common variants (>10% freq.) still showed a perturbation effect in close to 10% of the tested examples. These data sustain the estimate that >1,000 missense variants per individual genome may have an interaction perturbation effect. Whether these perturbations result in cellular phenotypes remains an open question.

These studies suggest that interaction disruptive coding variation is surprisingly widespread and that individual variation does affect protein interaction patterns. This can be particularly relevant when assessing cancer mutations for their potential impact on disease pathogenesis. Therefore, a pivotal next generation goal in interactome mapping is to assay all possible variants for their effect on protein interactions [147, 158, 159]. Deep mutagenesis approaches [12] in combination with stringent protein interaction assays in yeast, provide comprehensive mutational profiles about selected interaction pairs. The key idea is to use deep mutagenesis pools that at best contain all single amino acid variants of a protein and test for selective protein interaction perturbation. Enrichment of interacting or non-interacting protein pairs is for example achieved through yeast growth selection or fluorescence sorting (**Figure 2D and 3A-B**). Enrichment scores for each and every variant can be determined that indicate whether substitutions have an effect on protein binding or not [160], effectively creating an interaction-centric variant catalog for a protein.

With these catalogs in hand various analytical routes have been taken. One of the first studies combined mutational scanning data obtained in yeast with natural variation to study evolutionary paths of deleterious substitutions in the Pab1[RRM2]-eIF4G interaction [161]. Another, bacterial-2H based study compared hRAS mutational Raf interaction perturbation pattern with evolutionary sequence variation in the vertebrate lineage [162]. As described above, using deep mutagenic pools of BRCA1, Starita *et al*. assayed BARD1 binding capacities in a Y2H analysis in parallel to E3 ligase-dependent autoubiquitination activities. These data fed into models to predict the capacities of full-length BRCA1 variants in homology-directed DNA repair [142]. While individual protein interaction pairs have been mapped through deep mutagenesis approaches, a study focusing on several interactions in a large human protein complex demonstrated that deep mutagenesis can be performed at a larger scale. A sensitive reverse Y2H-based ‘off switch' for positive selection of interaction-disruptive variants through yeast growth from deep mutagenesis libraries was developed with the perspective to go beyond the single pair protein domain analyses. More than 1,000 interaction-disrupting amino acid mutations across eight subunits and nine interactions of the 0.5 MDa cilial BBSome complex were defined [163]. The network did support the elucidation of high-resolution structure of the complex [164], while the variant perturbation information can inform the functional assessment of BBSome disease mutations mapped in Bardet-Biedl syndrome [163]. However, deep mutagenesis approaches also show the capacity to move beyond assigned single amino acid mutations to the assessment of epistatic combinations of mutations. In yeast, more than 120,000 pairs of point mutations in the leucine zipper FOS and JUN interaction were subjected to selection through protein complementation and deep sequencing. The results demonstrated how physical interactions quantitatively relate to genetic interactions i.e. how two single mutations that affect the PPI combine [165]. The relative fraction of double mutant pairs that weaken (negative interaction) or strengthen (positive interaction) the FOS-JUN leucine zipper interaction is around 5% with a prevalence for cis genetic interactions and strongly determined by proximity in the PPI interface.

While the large-scale PPI mapping task as such [166, 167] is distributed between mass spectrometry based approaches in human cell lines [168] and Y2H techniques [169], the next frontier – studying the effects of coding variation on molecular interactions – is currently more or less exclusively approached with yeast PPI assays.

### SARS-Cov-2 spike protein variants modulate ACE2 receptor and antibody interaction

Recently, yeast genetics in combination with deep mutational scanning was the basis of outstanding research approaches related to the SARS-CoV-2 virus pandemic (**Figure 3B**). Starr *et al*. used yeast surface display for characterizing the SARS-CoV-2 spike protein interaction with the human receptor ACE2 [170] and human antibodies [171]. The receptor binding domain (RBD, aa 331-531) of spike protein, which mediates binding to ACE2 receptor, can be expressed on the yeast cell surface. The Bloom group integrated such a yeast protein-display system with deep mutational scanning to determine how each and every amino acid mutation in the SARS-CoV-2 spike-RBD impacts expression and its binding affinity for human ACE2 using fluorescently labeled RBD and ACE2 respectively. Intriguingly, this protein interaction deep mutational scanning approach provided a detailed molecular picture of variants that influence the RBD-ACE2 interaction and revealed the N501F and N501Y amino acid substitutions among the top four variants that strongly increase RBD-ACE2 affinity [170]. The latter spike protein variant, known as B.1.1.7 SARS-CoV-2 strain, indeed spread worldwide. The deeply mutagenized spike-RBD pool displayed on yeast was also employed to map antibody interactions, both to characterize commercially therapeutic antibodies such as the REGN-COV2 and Ly-CoV016 antibodies, as well as antibody binding from human sera. The mutational scanning readout of the antibody interactions comprehensively characterizes antibody escape mutations in the spike RBD domain (such as E406W or F486I) which may be important in understanding how mutations observed during viral surveillance impact the efficacy of antibody treatments or the immune response to vaccines.

## CONCLUSION

Through continuous improvement of yeast systems and substantial advances in genome scale screening approaches, yeast serves as a prime tool in molecular biology and human genetics. The rapid adaption to urgent research questions like in the current SARS-CoV-2 pandemic highlights its flexibility and broad, almost universal applicability in various fields of research. Here we emphasized deep mutational scanning approaches in yeast to comprehensively characterize coding variation in particular amino acid substitutions of unknown significance in cancer proteins. These protein activities can be conveniently selected because the activity is *absent* in yeast nevertheless causing a phenotype. On the other hand, using deep mutational scanning in protein interaction research provides a uniform, more general approach to study the impact of genetic variation on cellular protein function. However, mutations can impact several features of proteins. Therefore, an integrated combination of different yeast assays has the potential to fully characterize coding variation. Various features of proteins have been exploited in deep mutational scanning approaches quantitively assaying protein folding/abundance [172, 173], interaction [142, 163], aggregation [14], enzymatic activity [44], degradation/stability [174], transcriptional activity [126] and phase transition [14]. The combination of broadly applicable yeast assays will lead to quantitative characterization of all single mutant variants of human proteins, bridging the gap between genomic variation and protein function. Moreover, quantifying the activity of single- and double-mutant variants of a protein has revealed epistatic relationships. Interestingly, epistatic relationships between mutations, i.e. combinations of mutations that show a quantitative yeast readout different to the model prediction of the combined single mutant variants, were demonstrated to provide enough information to determine the three-dimensional backbone structure of the protein *de novo* [175, 176]. Deep mutational scanning approaches coupled to combinations of yeast phenotypic selection clearly show new directions for a comprehensive and quantitative biology of human protein function.

## Abbreviations:

APP: – amyloid precursor protein;
DNMT: – DNA-methyltransferase;
EGFR: – epidermal growth factor receptor;
FASAY: – functional analysis of separated alleles of p53 in yeast;
GAP: – GTPase-activating protein;
GPCR: – G protein coupled receptor;
HR: – homologous recombination;
MMS: – methyl methane-sulfonate;
MNNG: – N-methyl-N'-nitro-N-nitrosoguanidine;
NRTK: – non-receptor tyrosine kinase;
PARP: – poly(ADP-ribose) polymerase;
PHE: – 6(5H)-phenanthridinone;
PI3K: – posphoinositide-3-kinase;
PIP_2_: – phosphatidylinositol 4,5-bisphosphate;
PIP_3_: – phosphatidylinositol (3,4,5)-trisphosphate;
PPI: – protein-protein interaction;
PTK: – protein tyrosine kinase;
pY: – phospho-tyrosine;
RBD: – receptor binding domain;
RGS: – regulator of G protein signaling;
SAM: – S-adenosyl methionine;
WT: – wild type;
Y2H: – yeast two hybrid.
